# Associations between environmental heavy metals exposure and preserved ratio impaired spirometry in the U.S. adults

**DOI:** 10.1007/s11356-023-29688-y

**Published:** 2023-09-25

**Authors:** Chen Chen, Shunan Zhang, Ting Yang, Chen Wang, Guiling Han

**Affiliations:** 1 National Center for Respiratory MedicineNational Clinical Research Center for Respiratory DiseasesInstitute of Respiratory MedicineDepartment of Traditional Chinese Medicine for Pulmonary Diseases, Center of Respiratory Medicine, Chinese Academy of Medical Sciences, China-Japan Friendship Hospital, Beijing, 100029 People’s Republic of China; 2 National Center for Respiratory MedicineNational Clinical Research Center for Respiratory DiseasesInstitute of Respiratory MedicineDepartment of Pulmonary and Critical Care Medicine, Center of Respiratory Medicine, Chinese Academy of Medical Sciences, China-Japan Friendship Hospital, Beijing, 100029 People’s Republic of China; 3 Chinese Academy of Medical Sciences and Peking Union Medical College, Beijing, 100730 People’s Republic of China

**Keywords:** Chronic obstructive pulmonary disease, Preserved ratio impaired spirometry, Environmental pollutants, Heavy metals, Lung function, National Health and Nutrition Survey

## Abstract

We examined 9556 individuals aged 18 to 79 years who had information on spirometry testing and heavy metals and used multivariable logistic or linear regression to evaluate associations between serum levels of cadmium, lead, and mercury and PRISm and lung function in U.S. adults, which were conducted first in all participants, and then separately in never/former smokers and current smokers. The overall prevalence of PRISm was 7.02%. High levels of serum cadmium were significantly associated with PRISm in all individuals, no matter in never/former smokers (quartile 4 *vs* 1, the OR = 2.517, 95% CI = 1.376–4.604, *p*-trend = 0.0077) and current smokers (quartile 4 *vs* 1, the OR = 2.201, 95% CI = 1.265–3.830, *p*-trend = 0.0020). Serum lead and mercury were not significantly correlated with PRISm, regardless of smoking status. Serum cadmium was strongly correlated with lower FEV_1_/FVC, regardless of smoking status. Besides, serum cadmium was also significantly related to lower FVC % predicted in never/former smokers and lower FEV_1_% predicted in current smokers. Serum lead was strongly correlated with lower FVC % predicted and FEV_1_/FVC in all individuals and never/former smokers. And serum mercury was significantly associated with decrements in FVC % predicted in all individuals and current smokers. These findings demonstrate that serum cadmium is associated with a higher risk of PRISm and lower lung function, with the most significant effect on FEV_1_/FVC in particular. Our results also indicate that exposure to lead and mercury negatively affects lung function in never/former smokers and current smokers, respectively.

**Supplementary Information:**

The online version contains supplementary material available at 10.1007/s11356-023-29688-y.

## Introduction

Chronic obstructive pulmonary disease (COPD), the third greatest cause of death in the world (World Health Organization 2019), is considered a heterogeneous lung condition characterized by persistent airflow limitation due to pathological changes in the large and small airways (Global Initiative for Chronic Obstructive Lung Disease 2023), and lung function is the main diagnostic criterion. With a greater understanding of COPD, a more comprehensive diagnosis should be used to identity mild disease before permanent pathological alterations take place. The term preserved ratio impaired spirometry (PRISm), defined as a normal or preserved forced expiratory volume in 1 s (FEV_1_) /forced vital capacity (FVC) ratio (≥ 0.70) but a FEV_1_ of less than 80% predicted, has recently been proposed. Previous studies have shown that nearly half of individuals with PRISm may transition to COPD within the next 5 years (Wan et al. 2018; Wijnant et al. 2020) and that PRISm is associated with increased respiratory symptoms (Wan et al. 2014; Guerra et al. 2017), COPD-related comorbidities (cardiac disease, diabetes, obesity) (Heo et al. 2020; Jankowich et al. 2018; Park et al. 2018), and all-cause mortality (Wan et al. 2018, 2021; Higbee et al. 2022). Therefore, identifying the risk factors for PRISm is the key to the prevention and control of COPD, which includes smoking (Wan et al. 2014; Guerra et al. 2017), advanced age (Wan et al. 2014), female (Higbee et al. 2022; Wan et al. 2021), abnormal body weight (Guerra et al. 2017; Higbee et al. 2022; Wan et al. 2021), trunk fat mass and percentage (Higbee et al. 2022), asthma (Higbee et al. 2022), and diabetes (Wan et al. 2014; Mannino et al. 2012).

In addition to tobacco smoke, the roles of environmental pollutants in the pathogenesis of COPD are increasingly being emphasized, which may impair airway epithelial barrier function and subsequently lead to uncontrolled chronic inflammation, oxidative stress, and airway remodeling, resulting in persistent damage to the respiratory tract (Stolz et al. 2022; Aghapour et al. 2022). Among them, the health risks of environmental heavy metals are well known (Song and Li 2015; Manisalidis et al. 2020; Zhang et al. 2021). Although several observational studies have demonstrated the relationship between heavy metals exposure and lung diseases, however, such studies have mainly focused on the effects of heavy metals exposure levels on respiratory symptoms, lung function, hospitalization, and mortality in patients with a definite diagnosis of COPD (Wu et al. 2019; Jiang et al. 2022; Barry and Steenland 2019) ^[18–20]^, while a clear characterization of the absolute risk of early lung diseases in adults due to heavy metals exposure is lacking. Epidemiological data pointing to risk factors in early life suggests that poor lung health in adulthood may be partially caused by suboptimal growth and development (Portas et al. 2020). Higher blood levels of heavy metals are linked to lower pulmonary parameters in children, according to several analyses of clinical data (Zeng et al. 2017; Pan et al. 2020; Rosa et al. 2022). This could be a contributing factor to the rapid decline in lung function in adulthood.

To evaluate the effect of the heavy metals exposure determined by serum cadmium, lead, and mercury levels on the risk of early lung diseases (PRISm) and lung function impairment, we employed data reflecting U.S. adults (≥ 18 years) in this context. We hypothesized that exposure to heavy metals is linked to a higher risk of PRISm and lower lung function.

## Materials and methods

### Study population

A representative sample of the U.S. population was chosen for the National Health and Nutrition Examination Assessment (NHANES), a continuous cross-sectional survey of the health and nutritional status of the noninstitutionalized civilian population in the U.S. The NHANES protocols were approved by the institutional review boards (IRBs) of the CDC and NCHS, and all participants provided their informed permission. The following website provided information on NHANES protocols, methods, and IRB approval: https://www.cdc.gov/nchs/nhanes/index.htm.

**Fig. 1:**
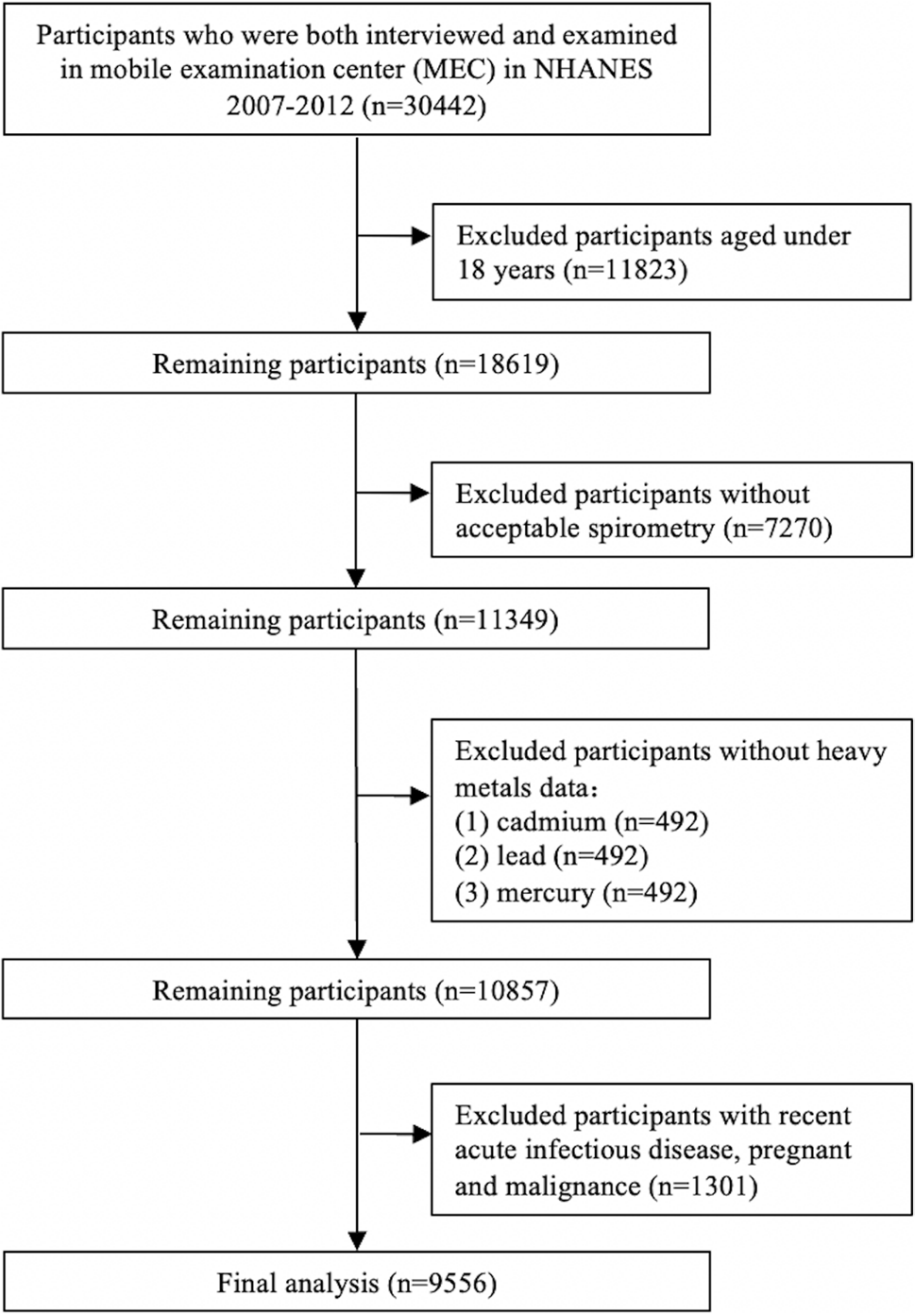
Flow diagram of study participants

In the 2007–2008, 2009–2010, and 2011–2012 NHANES cycles, 18,619 participants aged ≥ 18 years were surveyed. There were 10,857 participants with valid lung function and heavy metals data. Participants with recent acute infectious disease, pregnant, malignance, and other conditions were excluded. In the end, 9556 participants in total were enrolled in this study (Fig. 1).

### Spirometry

Lung function testing followed the recommendations of the American Thoracic Society. Participants in the NHANES from 2007 to 2012, aged 6 to 79 years, were invited to participate in spirometry. A more comprehensive description of inclusion and exclusion criteria were provided by the NHANES protocol (https://www.cdc.gov/nchs/data/nhanes/nhanes_11_12/spirometry_procedures_manual.pdf). PRISm was defined as individuals with spirometry with FEV_1_/FVC ≥ 0.70 and FEV_1_ < 80% predicted, obstructive spirometry was defined as individuals with spirometry with FEV_1_/FVC < 0.7, and normal spirometry was defined as individuals with spirometry with FEV_1_/FVC ≥ 0.70 and FEV_1_ ≥ 80% predicted.

### Exposure history

Cadmium (Cd), serum lead (Pb), and mercury (Hg) concentrations were determined using inductively coupled plasma mass spectrometry (PerkinElmer, Norwalk, Conn). Further information on the examination process and methodology could be found online (https://wwwn.cdc.gov/Nchs/Nhanes/2011–2012/PBCD_E.htm).

The questionnaire provided information on smoking and occupational exposure. Smoking status was defined as never, former, or current smoking. Participants who answered “No” about the question “Have you smoked at least 100 cigarettes in your entire life?” were classified as “never smokers.” Based on information on the age of smoking initiation and cessation, cigarette consumption was computed in pack-year. 20 cigarettes smoked per day for a year were referred to as a pack-year. The participants were then divided into three groups based on pack-year: never-smokers, smokers with fewer than ten pack-years, and smokers with ten or more pack-years. By inhaling smoke, non-smokers who reside in or work among smokers are said to be engaging in passive smoking. A “Yes” response to the following questions constituted occupational exposure: “In any job, have you ever been exposed to dust, exhaust fumes, or any other gases, vapors or fumes?”.

### Other variables

Chronic lower respiratory disease and cardiovascular disease were self-reported according to the question about “Has a doctor or other health professional ever told you that you had emphysema, chronic bronchitis, asthma, coronary heart disease, congestive heart failure, and stroke?”. We used the question about “How old were you when you were first told had emphysema, bronchitis or asthma?” to define the history of respiratory diseases in children before the age 14 years. Hypertension was defined as a self-reported diagnosis of hypertension or self-reported use of antihypertensive medication. Diabetes was defined as a self-reported diagnosis of diabetes or self-reported use of insulin or hypoglycemic medications.

As part of the NHANES Household Questionnaire Interview, only those ≥ 40 years were questioned about respiratory symptoms. The presence of chronic cough, phlegm production, wheezing, and shortness of breath is determined by the each of the following 4 questions: “Do you usually cough on most days for 3 consecutive months or more during the year?", “Do you bring up phlegm on most days for 3 consecutive months or more during the year?”, “In the past 12 months have you had wheezing or whistling in your chest?”, and “Have you had shortness of breath either when hurrying on the level or walking up a slight hill?”.

Questionnaires were also used to collect data on age, sex, race/ethnicity, BMI, educational level, family income (poverty income ratio, PIR), insurance coverage, sedentary activity minutes per day.

### Statistical analysis

SAS 9.4 was used to process all data, and a two-sided significance level of *p* < 0.05 was used. Continuous variables were presented as mean ± standard deviation (SD). When the data exhibited a normal distribution and homogeneity of variance, the independent-sample t test was applied. When the data did not meet the condition, Wilcoxon testing was utilized. The Chi-square test was used to compare categorical variables that were provided as frequencies (percentages).

We applied multivariable logistic regression models to investigate the association between heavy metals (i.e., cadmium, lead, and mercury) and PRISm. The following variables were adjusted after the univariate analysis because they were statistically and clinically significant: age, gender, race/ethnicity, BMI, PIR, health insurance, sedentary activity, childhood diseases history, diabetes, and occupational exposure. We carried out a sensitivity analysis, as seen below, to more confidently deduce the statistical of analysis findings in research. To check for a non-linear association, we first converted heavy metals into a categorical variable (quartile). Following that, linear trends were performed to confirm the outcomes of using heavy metals as a continuous variable.

To explore the association between heavy metals and lung function, we used multivariable linear regression model, which adjusted for age, gender, race/ethnicity, BMI, PIR, health insurance, sedentary activity, childhood diseases history, diabetes, hypertension, and occupational exposure. And a subgroup analysis of the participants was carried out based on smoking status (never/former smokers or current smokers).

## Results

### Prevalence and correlates of PRISm

11,349 (61.00%) of the 18,619 participants aged 18 to 79 years who had valid lung function during NHANES 2007–2012. 1476 of these participants had missing information on serum heavy metals were excluded. 1301 of these participants with recent acute infectious disease, pregnant and malignance; thus, a total of 9556 individuals (mean age 44.54 ± 16.76 years, 48.46% women, 50.07% never-smokers) were included in the final analysis. Table 1 provided information on the demographics and general traits of the study population.

**Table 1:** Characteristics of study participants, NHANES 2007–2012

Characteristics	PRISm (*n* = 671)	Normal spirometry (*n* = 7653)	*p*-Value (PRISm vs Normal spirometry)	Obstructive spirometry (*n* = 1232)	*p*-Value (PRISm vs Obstructive spirometry)
Demographics					
Age group			< 0.0001		< 0.0001
< 30 y	109 (16.24)	2060 (26.92)		71 (5.76)	
30–39 y	96 (14.31)	1535 (20.06)		111 (9.01)	
40–49 y	120 (17.88)	1460 (19.08)		181 (14.69)	
50–59 y	149 (22.21)	1142 (14.92)		272 (22.08)	
60–69 y	137 (20.42)	971 (12.69)		330 (26.79)	
70–79 y	60 (8.94)	485 (6.34)		267 (21.67)	
Sex			0.6542		< 0.0001
Female	333 (49.63)	3867 (50.53)		431 (34.98)	
Male	338 (50.37)	3786 (49.47)		801 (65.02)	
Race/ethnicity			< 0.0001		< 0.0001
Non-Hispanic White	107 (15.95)	1777 (23.22)		115 (9.33)	
Non-Hispanic Black	318 (47.39)	3796 (49.60)		821 (66.64)	
Mexican American	246 (36.66)	2080 (27.18)		296 (24.03)	
BMI (kg/m^2^)			< 0.0001		< 0.0001
< 18.5 (underweight)	20 (2.99)	96 (1.26)		30 (2.44)	
18.5–24.9 (normal weight)	110 (16.44)	2089 (27.31)		412 (33.50)	
25–29.9 (overweight)	158 (23.62)	2556 (33.42)		445 (36.18)	
≥ 30 (obese)	381 (56.95)	2907 (38.01)		343 (27.89)	
Missing	2	5		2	
Educational level			< 0.0001		0.8612
Primary school and less	65 (10.19)	637 (8.93)		123 (10.12)	
Middle and high school	299 (46.87)	2712 (38.01)		555 (45.64)	
College and higher	274 (42.95)	3786 (53.06)		538 (44.24)	
Missing	33	518		16	
PIR			< 0.0001		0.0024
< 1.85	340 (53.71)	3144 (44.55)		530 (46.21)	
≥ 1.85	293 (46.29)	3914 (55.45)		617 (53.79)	
Missing	38	595		85	
Health insurance coverage			0.1441		0.0085
No	173 (25.82)	2176 (28.47)		253 (20.55)	
Yes	497 (74.18)	5467 (71.53)		978 (79.45)	
Missing	1	10		1	
Sedentary activity			0.0131		0.0357
< 3 h	120 (17.91)	1584 (20.72)		217 (17.66)	
3-6 h	231 (34.48)	2861 (37.43)		494 (40.20)	
≥ 6 h	319 (47.61)	3198 (41.84)		518 (42.15)	
Missing	1	10		3	
Exposure history					
Smoking status			< 0.0001		< 0.0001
Never smoker	303 (47.49)	4143 (58.09)		339 (27.88)	
Former smoker	160 (25.08)	1470 (20.61)		395 (32.48)	
Current smoker	175 (27.43)	1519 (21.30)		482 (39.64)	
Missing	33	521		16	
Smoking exposure (pack-years)			< 0.0001		< 0.0001
0	303 (48.79)	4143 (59.59)		339 (28.80)	
< 10	105 (16.91)	1571 (22.60)		229 (19.46)	
≥ 10	213 (34.30)	1238 (17.81)		609 (51.74)	
Median (IQR)	16.9 (28.03)	7.6 (16.8)		22 (34)	
Missing	50	701		55	
Passive smoking			0.8658		0.9268
No	204 (84.30)	2941 (83.88)		363 (84.03)	
Yes	38 (15.70)	565 (16.12)		69 (15.97)	
Missing	429	4147		800	
Smokers living in the home			< 0.0001		0.0622
0	158 (47.88)	2541 (66.92)		251 (39.97)	
1–2	153 (46.36)	1102 (29.02)		337 (53.66)	
≥ 3	19 (5.76)	154 (4.06)		40 (6.37)	
Missing	341	3856		604	
Mineral dusts			0.1995		0.0033
No	419 (65.78)	4991 (68.25)		711 (58.76)	
Yes	218 (34.22)	2322 (31.75)		499 (41.24)	
Missing	34	340		22	
Organic dusts			0.4042		0.0410
No	483 (75.82)	5657 (77.27)		868 (71.38)	
Yes	154 (24.18)	1664 (22.73)		348 (28.62)	
Missing	34	332		16	
Fumes from machinery or engines			0.4509		< 0.0001
No	487 (76.33)	5492 (74.99)		780 (64.09)	
Yes	151 (23.67)	1832 (25.01)		437 (35.91)	
Missing	33	329		15	
Any other gases, vapors or fumes			0.4071		0.0034
No	426 (66.77)	5007 (68.36)		728 (59.82)	
Yes	212 (33.23)	2317 (31.64)		489 (40.18)	
Missing	33	329		15	
Medical history/Comorbidities					
Emphysema, bronchitis or asthma during childhood			0.0013		0.1292
No	475 (70.79)	5842 (76.34)		912 (74.03)	
Yes	196 (29.21)	1811 (23.66)		320 (25.97)	
Emphysema			0.0004		0.0012
No	628 (98.28)	7097 (99.45)		1153 (95.29)	
Yes	11 (1.72)	39 (0.55)		57 (4.71)	
Missing	32	517		22	
Chronic bronchitis			< 0.0001		0.4515
No	586 (91.85)	6864 (96.24)		1096 (90.80)	
Yes	52 (8.15)	268 (3.76)		111 (9.20)	
Missing	33	521		25	
Asthma			< 0.0001		0.0684
No	548 (81.67)	6735 (88.07)		961 (78.13)	
Yes	123 (18.33)	912 (11.93)		269 (21.87)	
Missing	0	6		2	
Close relative had asthma			0.0578		0.0826
No	506 (76.67)	6019 (79.77)		962 (80.10)	
Yes	154 (23.33)	1526 (20.23)		239 (19.90)	
Missing	11	108		31	
Hypertension			< 0.0001		0.5455
No	394 (58.98)	5638 (73.73)		709 (57.55)	
Yes	274 (41.02)	2009 (26.27)		523 (42.45)	
Missing	3	6		0	
Coronary heart disease			< 0.0001		0.3840
No	602 (94.80)	7008 (98.29)		1135 (93.80)	
Yes	33 (5.20)	122 (1.71)		75 (6.20)	
Missing	36	523		22	
Heart failure			< 0.0001		0.7058
No	608 (95.75)	7052 (98.91)		1152 (95.36)	
Yes	27 (4.25)	78 (1.09)		56 (4.64)	
Missing	36	523		24	
Stroke			< 0.0001		0.6390
No	613 (95.93)	7015 (98.40)		1157 (95.46)	
Yes	26 (4.07)	114 (1.60)		55 (4.54)	
Missing	32	524		20	
Diabetes			< 0.0001		0.0004
No	527 (80.34)	6905 (91.77)		1044 (86.57)	
Yes	129 (19.66)	619 (8.23)		162 (13.43)	
Missing	15	129		26	
Symptoms (≥ 40 y)					
Chronic cough			0.0017		0.0316
No	410 (87.98)	3737 (92.20)		879 (83.71)	
Yes	56 (12.02)	316 (7.80)		171 (16.29)	
Missing	205	3600		182	
Coughing phlegm			0.0014		0.0049
No	420 (90.13)	3811 (93.96)		890 (84.76)	
Yes	46 (9.87)	245 (6.04)		160 (15.24)	
Missing	205	3597		182	
Wheezing			< 0.0001		0.0117
No	535 (79.73)	6851 (89.61)		919 (74.59)	
Yes	136 (20.27)	794 (10.39)		313 (25.41)	
Shortness of breath			< 0.0001		0.0619
No	256 (54.94)	2984 (73.61)		630 (60.06)	
Yes	210 (45.06)	1070 (26.39)		419 (39.94)	
Missing	205	3599		183	
Lung function					
FEV_1_% predicted	72.80 ± 6.68	100.69 ± 11.39	< 0.0001	80.02 ± 17.68	< 0.0001
FVC % predicted	77.07 ± 14.42	104.31 ± 17.87	< 0.0001	102.58 ± 21.49	< 0.0001
FEV_1_/FVC	0.78 ± 0.06	0.81 ± 0.06	< 0.0001	0.63 ± 0.07	< 0.0001
Serum heavy metals (μg/L)					
Cadmium	0.65 ± 0.74	0.47 ± 0.54	< 0.0001	0.84 ± 0.93	< 0.0001
Lead	1.62 ± 1.81	1.51 ± 1.87	0.0003	2.12 ± 1.50	< 0.0001
Mercury	1.39 ± 2.16	1.37 ± 2.04	0.1294	1.33 ± 1.83	0.5378

Results are shown as N (%) for binary variables, and as mean ± standard deviation (SD) for continuous variables. *BMI*, body mass index;* PIR*: poverty income ratio; *FEV*_*1*_, forced expiratory volume in 1 s; *FVC*, forced vital capacity; *PRISm*, preserved ratio impaired spirometry; *NHANES*, National Health and Nutrition Examination Survey

671 (7.02%) of 9556 individuals had PRISm, 1232 (12.89%) had obstructive spirometry, and 7653 (80.09%) individuals with normal spirometry. Age analysis showed that participants with PRISm tends to be older than those participants with normal spirometry and accounted for a higher proportion of participants over 50 years old (346 [51.56%] of 671 participants *vs* 2598 [33.95%] of 7653 participants). The prevalence of PRISm was 6.33% among individuals who self-reported as never smoker, 7.90% among former smoker individuals, and 8.04% among current smoker individuals. There was a statistically significant difference in smoking status between individuals with PRISm and normal spirometry, including a higher proportion of smokers (335 [52.51%] of 638 individuals *vs* 2989 [41.91%] of 7132 individuals), a higher median [IQR] number of pack-years (0.25 [18.00] *vs* 0.00 [4.65]), and more smokers in the home (172 [52.12%] of 330 individuals *vs* 1256 [33.08%] of 3797 individuals) among individuals with PRISm.

We also found that Mexican American (246 [36.66%] of 671 individuals *vs* 2080 [27.18%] of 7653 individuals), obesity (381 [56.95%] of 669 individuals *vs* 2907 [38.01%] of 7648 individuals), lower levels of education (364 [57.06%] of 638 individuals *vs* 3349 [46.94%] of 7135 individuals), lower household income (340 [53.71%] of 633 individuals *vs* 3144 [44.55%] of 7058 individuals), long periods of sedentary behavior (319 [47.61%] of 670 individuals *vs* 3198 [41.84%] of 7643 individuals) were more prevalent in participants with PRISm than normal spirometry participants. Additionally, statistically significant differences were found between PRISm individuals and normal spirometry individuals in terms of comorbidities (respiratory, cardiovascular, and metabolic), respiratory symptoms (chronic cough, coughing up phlegm, wheezing, dyspnea), and lung function. Participants with PRISm had higher rates of being Mexican American and Non-Hispanic White (353 [52.61%] of 671 participants compared to 707 [33.36%] of 1232 participants), obesity (381 [56.95%] of 669 participants compared to 343 [27.89%] of 1230 participants), lower household income (340 [53.71%] of 633 participants compared to 530 [46.21%] of 1147 participants), and having diabetes (129 [19.66%] of 656 participants *vs* 162 [13.43%] of 1206 participants) than participants with airflow obstruction.

The levels of serum cadmium (0.65 ± 0.74 *vs* 0.47 ± 0.54, *p* < 0.0001) and lead (1.62 ± 1.81 *vs* 1.51 ± 1.87, *p* = 0.0003) were higher in individuals with PRISm than in those with normal spirometry, except that there was no significant difference in serum mercury.

### Association between heavy metals and PRISm

Tables 2, 3 and 4 displayed the relationships between PRISm and heavy metals (serum cadmium, lead, and mercury). The outcomes of multivariable analyses of serum cadmium and PRISm were presented in Table 2. In the analysis of all subjects, those with the highest (fourth) quartile of serum cadmium had 1.695 times higher odds of PRISm than those with the lowest (first) quartile of serum cadmium (quartile 4 *vs* 1, the OR = 1.695, 95% CI = 1.293–2.221, *p*-trend < 0.0001). After stratification based on smoking status, there was strong association between serum cadmium and PRISm in never/former smokers. Subjects with serum cadmium levels above the first quartile had 2.088 to 2.517 times greater higher odds of PRISm than those with serum cadmium levels in the first (lowest) quartile (quartile 2 *vs* 1, the OR = 2.088, 95% CI = 1.162–3.750; quartile 3 *vs* 1, the OR = 2.314, 95% CI = 1.265–4.234; quartile 4 *vs* 1, the OR = 2.517, 95% CI = 1.376–4.604, *p*-trend = 0.0077). The odds of PRISm were 2.201 times greater among current smokers whose serum cadmium levels were in the highest (fourth) quartile compared to the lowest (first) quartile (quartile 4 *vs* 1, the OR = 2.201, 95% CI = 1.265–3.830, *p*-trend = 0.0020). The results of the influence of serum lead and mercury on PRISm by multivariable analyses showed that serum lead and mercury were not strongly correlated with PRISm, either in all individuals, never/former or current smokers (Tables 3 and 4). Furthermore, the results of subgroup analyses indicated that the association between the higher level of serum lead and PRISm was more pronounced among those who tend to be younger (under 30 years), self-identified as non-Hispanic Black, were normal weight, had occupational exposure to exhaust fumes from machinery or engines, and did not have hypertension (Table S1). And among participants who self-reported as Mexican American or combined hypertension, the relationship between the higher serum mercury levels and PRISm was more apparent (Table S2).

**Table 2:** Association of serum cadmium with PRISm, NHANES 2007–2012^1^

Exposure	Serum cadmium (ug/L)
	Odds ratio (95% confidence interval)
All individuals (n = 8324)	
Quartile 1 (< 0.19 ug/L)	1.00
Quartile 2 (0.19–0.29 ug/L)	1.033 (0.768, 1.390)
Quartile 3 (0.29–0.52 ug/L)	1.302 (0.985, 1.720)
Quartile 4 (≥ 0.52 ug/L)	1.695 (1.293, 2.221) ^**^
*p*-trend	< 0.0001
Never/former smokers (n = 6076)	
Quartile 1 (< 0.14 ug/L)	1.00
Quartile 2 (0.14–0.26 ug/L)	2.088 (1.162, 3.750) ^*^
Quartile 3 (0.26–0.37 ug/L)	2.314 (1.265, 4.234) ^**^
Quartile 4 (≥ 0.37 ug/L)	2.517 (1.376, 4.604) ^**^
*p*-trend	0.0077
Current smokers (n = 1694)	
Quartile 1 (< 0.57 ug/L)	1.00
Quartile 2 (0.57–0.93 ug/L)	1.483 (0.835, 2.633)
Quartile 3 (0.93–1.45 ug/L)	1.478 (0.830, 2.633)
Quartile 4 (≥ 1.45 ug/L)	2.201 (1.265, 3.830) ^**^
*p*-trend	0.0020

*BMI*, body mass index; *PIR*: poverty income ratio; *PRISm*, preserved ratio impaired spirometry; *NHANES*, National Health and Nutrition Examination Survey. ^1^ All models were adjusted: age, gender, race/ethnicity, BMI, PIR, health insurance, sedentary activity, history of childhood diseases (emphysema, bronchitis, or asthma), diabetes, and occupational exposure to mineral dusts, organic dusts or exhaust fumes. Subgroup analysis according to smoking status. *p*-Value: ^*^ *p* < 0.05, ^**^ *p* < 0.01

**Table 3:** Association of serum lead with PRISm, NHANES 2007–2012^1^

Exposure	Serum lead (ug/dL)
	Odds ratio (95% confidence interval)
All individuals (n = 8324)	
Quartile 1 (< 0.73 ug/dL)	1.00
Quartile 2 (0.73- 1.12 ug/dL)	1.026 (0.778, 1.354)
Quartile 3 (1.12- 1.75 ug/dL)	1.180 (0.894, 1.556)
Quartile 4 (≥ 1.75 ug/dL)	1.105 (0.827, 1.476)
*p*-trend	0.3833
Never/former smokers (n = 6076)	
Quartile 1 (< 0.72 ug/L)	1.00
Quartile 2 (0.72–1.10 ug/L)	1.061 (0.772, 1.458)
Quartile 3 (1.10–1.68 ug/L)	1.090 (0.786, 1.513)
Quartile 4 (≥ 1.68 ug/L)	1.141 (0.814, 1.601)
*p*-trend	0.3522
Current smokers (n = 1694)	
Quartile 1 (< 0.97 ug/L)	1.00
Quartile 2 (0.97–1.46 ug/L)	1.050 (0.637, 1.732)
Quartile 3 (1.46–2.19 ug/L)	0.847 (0.500, 1.435)
Quartile 4 (≥ 2.19 ug/L)	0.742 (0.422, 1.307)
*p*-trend	0.0812

*BMI*, body mass index; *PIR*: poverty income ratio; *PRISm*, preserved ratio impaired spirometry; *NHANES*, National Health and Nutrition Examination Survey. ^1^ All models were adjusted: age, gender, race/ethnicity, BMI, PIR, health insurance, sedentary activity, history of childhood diseases (emphysema, bronchitis, or asthma), diabetes, and occupational exposure to mineral dusts, organic dusts or exhaust fumes. Subgroup analysis according to smoking status

**Table 4:** Association of serum mercury with PRISm, NHANES 2007–2012^1^

Exposure	Serum mercury (ug/L)
	Odds ratio (95% confidence interval)
All individuals (n = 8324)	
Quartile 1 (< 0.44 ug/L)	1.00
Quartile 2 (0.44—0.79 ug/L)	1.024 (0.800, 1.309)
Quartile 3 (0.79—1.50 ug/L)	0.828 (0.642, 1.068)
Quartile 4 (≥ 1.50 ug/L)	0.906 (0.701, 1.172)
*p*-trend	0.2642
Never/former smokers (n = 6076)	
Quartile 1 (< 0.47 ug/L)	1.00
Quartile 2 (0.47–0.86 ug/L)	0.782 (0.586, 1.043)
Quartile 3 (0.86–1.63 ug/L)	0.809 (0.606, 1.079)
Quartile 4 (≥ 1.63 ug/L)	0.854 (0.635, 1.150)
*p*-trend	0.3752
Current smokers (n = 1694)	
Quartile 1 (< 0.40 ug/L)	1.00
Quartile 2 (0.40–0.70 ug/L)	1.201 (0.743, 1.940)
Quartile 3 (0.70–1.29 ug/L)	1.050 (0.639, 1.727)
Quartile 4 (≥ 1.29 ug/L)	1.068 (0.645, 1.769)
*p*-trend	0.8742

*BMI*, body mass index; *PIR*: poverty income ratio; *PRISm*, preserved ratio impaired spirometry; *NHANES*, National Health and Nutrition Examination Survey. ^1^ All models were adjusted: age, gender, race/ethnicity, BMI, PIR, health insurance, sedentary activity, history of childhood diseases (emphysema, bronchitis, or asthma), diabetes, and occupational exposure to mineral dusts, organic dusts or exhaust fumes. Subgroup analysis according to smoking status

To investigate the potential combined effects of higher serum cadmium, lead, and mercury levels on PRISm, we carried out secondary analyses. (Figure S1). Individuals with high levels of both serum cadmium and serum lead had 1.59 times the risk of developing PRISm compared to those with low levels of both serum cadmium and serum lead, while those with high levels of serum cadmium but low levels of lead had 1.53 times the risk. Contrarily, individuals who had high lead levels but low cadmium levels had no significantly higher odds of PRISm than those who had low levels of both serum cadmium and serum lead. Individuals with high levels of serum cadmium and low levels of serum mercury exhibited 1.57 times significantly greater chances of PRISm than those with low levels of both serum cadmium and serum mercury after combining the two elements in the serum. Nevertheless, we did not discover a connection between high levels of serum cadmium and serum mercury and PRISm. Additionally, we found no evidence that the impact of combination of various serum lead and serum mercury levels on PRISm.

### Multivariable analysis of the heavy metals and lung function

Table 5 showed the results of the multivariable analysis of the heavy metals (serum cadmium, lead, and mercury) and lung function parameters. In this analysis, serum cadmium was strongly correlated with lower FEV_1_% predicted (β =  − 0.808, 95% CI =  − 1.097, − 0.519), FVC % predicted (β =  − 0.632, 95% CI =  − 1.026, − 0.237), and FEV_1_/FVC (β =  − 0.005, 95% CI =  − 0.006, − 0.004) among all participants. After stratification by smoking status, serum cadmium was significantly connected to the decreased FEV_1_/FVC in both never/former smokers and current smokers. In addition, serum cadmium exhibited a strong correlation with decrements in FVC % predicted (β =  − 1.151, 95% CI =  − 1.679, − 0.623) in never/former smokers and in FEV_1_% predicted (β =  − 1.449, 95% CI =  − 2.469, − 0.428) in current smokers. Serum lead was strongly connected to the lower FVC % predicted and FEV_1_/FVC in all participants and never/former smokers. Moreover, serum lead was significantly correlated with increments in FEV_1_% predicted in current smokers. Serum mercury was significantly connected to increments in FEV_1_% predicted and decrements in FVC % predicted, with similar but greater associations in current smokers. We found no significant relationship between serum mercury and FEV_1_/FVC, before or after stratification by smoking status.

**Table 5:** Heavy metals (serum cadmium, lead, and mercury) and lung function parameters, NHANES 2007–2012^1^

	Serum cadmium	Serum lead	Serum mercury
Parameters	β (95% confidence interval)		
All individuals (n = 8324)			
FEV_1_% predicted	-0.808 (-1.097, -0.519) ^**^	0.663 (-0.403, 0.257)	0.350 (0.062, 0.638)^*^
FVC % predicted	-0.632 (-1.026, -0.237) ^**^	-0.623 (-1.073, -0.174) ^**^	-0.438(-0.83, -0.045) ^*^
FEV_1_/FVC	-0.005 (-0.006, -0.004) ^**^	-0.003 (-0.004, -0.002) ^**^	0.000 (-0.001,0.001)
Never/former smokers (n = 6076)			
FEV_1_% predicted	-0.347 (-0.73,0.035)	0.018 (-0.358, 0.395)	0.182 (-0.143, 0.508)
FVC % predicted	-1.151 (-1.679, -0.623) ^**^	-0.760 (-1.281, -0.239) ^**^	-0.358 (-0.809, 0.092)
FEV_1_/FVC	-0.002 (-0.004, -0.001) ^**^	-0.002 (-0.003, 0.000) ^*^	-0.001 (-0.002, 0.001)
Current smokers (n = 1694)			
FEV_1_% predicted	-1.449 (-2.469, -0.428) ^**^	0.868 (0.106, 1.63) ^*^	0.700 (0.094, 1.306) ^*^
FVC % predicted	-1.272 (-2.596, 0.053)	-0.308 (-1.296, 0.681)	-0.821 (-1.607, -0.036)^*^
FEV_1_/FVC	-0.005 (-0.009, -0.001)^**^	0.000 (-0.003, 0.003)	0.002 (0.000, 0.005)

*BMI*, body mass index; *PIR*: poverty income ratio; *FEV*_*1*_, forced expiratory volume in 1 s; *FVC*, forced vital capacity; *NHANES*, National Health and Nutrition Examination Survey. ^1^ All models were adjusted for age, gender, race/ethnicity, BMI, PIR, health insurance, sedentary activity, history of childhood diseases (emphysema, bronchitis, or asthma), diabetes, hypertension, and occupational exposure to mineral dusts, organic dusts or exhaust fumes. Subgroup analysis according to smoking status. *p*-Value: ^*^ *p* < 0.05, ^**^ *p* < 0.01

## Discussion

To our knowledge, this is the first report of an association between PRISm and heavy metals. A positive significant relationship between serum cadmium and the prevalence of PRISm was found in this cross-sectional investigation of 9556 participants, and this relationship was more pronounced in never or former smokers than in current smokers. Serum cadmium was strongly correlated with lower FEV_1_/FVC, regardless of smoking status. Besides, serum cadmium was also significantly associated with lower FVC % predicted in never/former smokers and lower FEV_1_% predicted in current smokers. After adjusting for confounders, the relationship between the exposure variable and the outcome variables remained consistent. Although serum lead and mercury were not significantly connected with PRISm, either in all individuals, never/former or current smokers, subgroup analysis stratified by the age, sex, exposure history, and other variables revealed that this correlation could be applied to the population with different age, races, ethnicities, BMI, environmental pollutant exposure conditions and comorbidities. Serum lead was significantly associated with lower FVC % predicted and FEV_1_/FVC in all participants and never/former smokers. And serum mercury was significantly associated with decrements in FVC % predicted in all participants and current smokers.

We now realize that several overlapping risk factors affect COPD, which is a complicated and heterogeneous disease, as our understanding of it has progressively improved. Because smoking is not the only cause of COPD, it is important to emphasize that unchecked exposure to environmental pollutants over the course of a person’s lifetime is the main environmental risk factor (Stolz et al. 2022).

An accumulating heavy metal called cadmium (Cd) is naturally present in the Earth's crust (Centers for Disease Control and Prevention 2017). Cadmium is primarily ingested through food and inhaled through tobacco and environmental workplace exposure (including zinc smelters, battery manufacturing, vehicle radiators, and production units for paint and pigmen), adversely affecting immune function and lung host defense. Compared to other transition metals, cadmium has an extremely long resident half-life (10–30 years) in the body (Knoell and Wyatt 2021). Mechanisms of cadmium toxicity in the lung include the induction of oxidative stress, immune-mediated inflammatory reaction, disruption of barrier mechanisms, possible impaired DNA repair, endoplasmic reticulum stress and apoptosis, mitochondrial autophagy, and DNA methylation (Cirovic et al. 2022; Tao and Zhang 2018; Sundblad et al. 2016; Messner et al. 2016; Cao et al. 2021; Martin and Fry 2018). Cadmium will interfere with the normal function of other bivalent metal ions when it enters the body. For instance, Cd^2+^ inhibits the activity of antioxidant enzymes and has an impact on the removal of oxygen free radicals by directly interacting with the bivalent metal ions in antioxidant enzymes (Cirovic et al. 2022) ^[24]^. Innate immune cells such as macrophages, neutrophils, and dendritic cells, as well as mast cells, eosinophils, basophils, and natural killer cells, can be activated by excessive oxygen free radical disease, which can also cause IL-6, IL-8, TNF-α, TGF-β, and FOXP-3 to promote the release of inflammatory mediators (Knoell and Wyatt 2021; Tao and Zhang 2018; Sundblad et al. 2016). Through oxidative stress, Cd can also contribute to mitochondrial damage, which causes malfunction and mitochondrial autophagy (Messner et al. 2016; Cao et al. 2021); The methylation of functional gene promoter regions can be impacted by DNMT expression level interference, which will then have an impact on epigenetic (Martin et al. 2018). Additionally, cadmium has a direct impact on the adherence junction proteins, causing the synthesis of pulmonary MMP-2 and MMP-9, and accelerating collagen and elastin breakdown that results in emphysema (Sundblad et al. 2016; Kirschvink et al. 2005; Surolia et al. 2015). Multiple observational studies have demonstrated the relationship between acute or chronic cadmium exposure and lung diseases from a clinical perspective, including respiratory symptoms (Li et al. 2020; Yang et al. 2019), impaired lung function (Leem et al. 2015; Rokadia and Agarwal 2013), incidence of chronic airway disease (Rokadia and Agarwal 2013; Torén et al. 2019; Rahman et al. 2022a, b), and mortality (Yao et al. 2021; Park et al. 2020).

Tobacco is the main source of cadmium exposure (Satarug and Moore 2004). Compared to never smokers, cadmium concentrations in the blood of tobacco smokers can be up to 4 or 5 times higher (Ganguly et al. 2018; Agency for Toxic Substances and Disease Registry 2012). The research of Hassan et al. (Hassan et al. 2014) revealed significantly greater accumulation of cadmium in the lung of COPD GOLD stage IV patients compared to GOLD stage 0 patients, which was directly proportional to the total tobacco consumption (5 8 ± 10.8 pack-years *vs*. 22.5 ± 12.1 pack-years). Therefore, this study conducted stratified analysis based on whether the patients smoked or not, and the findings demonstrated that never or former smokers had a stronger positive significant relationship between serum cadmium and the prevalence of PRISm than did current smokers. Serum cadmium was also significantly associated with lower FEV_1_% predicted, FVC% predicted, and FEV_1_/FVC in all participants, with the most significant effect on FEV_1_/FVC in particularly, and regardless of the effect of smoking factor. In addition, serum cadmium was significantly connected with decrements in FVC% predicted in never/former smokers and in FEV_1_% predicted in current smokers. Among never smokers, occupational exposure to cadmium has been considered a risk factor for the development of emphysema and impaired lung function (Mannino et al. 2004; Davison et al. 1988; Balmes et al. 2003). In addition, male long-term smokers who were exposed to cadmium during 4 years of employment as furnace operators reported a rapid loss in lung function and a quicker development of emphysema (Leduc et al. 1993). It seems plausible that this effect on workers who are exposed to cadmium at work is caused by the increased cadmium deposition in the lungs brought on by tobacco smoking. This highlights the superimposed negative consequences of tobacco smoking and occupational cadmium exposure on the respiratory system.

A toxic heavy metal also present in the earth's crust is lead (Pb). (World Health Organization 2022). Airborne lead is deposited in soil and water. Thus, human exposure to lead occurs through the food chain, drinking water, and occupational exposure occurs in smelting plants, paint, glass, ceramic industries (Gomes et al. 2018). Previous epidemiological studies have indicated the associations of lead exposure with lung function impairment in both general population and occupational workers (Yang et al. 2019; Leem et al. 2015; Rokadia and Agarwal 2013; Gomes et al. 2018). Oxidative stress is a known mechanism underpinning lead (Matović et al. 2015), and numerous single nucleotide polymorphisms (SNPs) in genes associated to oxidative stress have been found to alter how air pollutants affect lung diseases (Castro-Giner et al. 2009; Thun et al. 2014; John et al. 2017; Kim et al. 2018). An occupational cohort study of 1243 workers in a coke-oven plant by Wei Wei et al. showed a considerable effect of lead exposure on the decline in FEV_1_, which was regulated by the *NQO1* rs2917670 genotypes (Wei et al. 2020). In our study, although we failed to find an association between serum lead exposure and the prevalence of PRISm in the whole population, the results of subgroup analyses indicated that the association between the higher level of serum lead and PRISm was more pronounced among those who tend to be younger (under 30 years), self-identified as non-Hispanic Black, were normal weight, had occupational exposure to exhaust fumes from machinery or engines, and did not have hypertension. The findings of this study emphasize the importance of exploring the potential mechanisms underlying the individual genetic susceptibility-environment interaction for the development of COPD. And a more in-depth investigation into these COPD subtypes is essential to develop precision COPD management.

Mercury (Hg) is a highly toxic heavy metal that primarily attacks the respiratory system in the form of mercury vapor (Mattila et al. 2021). Compared to most studies describing the impact of mercury on the neurological system, renal, skin, and reproductive system (Yang et al. 2020; Bjørklund et al. 2017), studies regarding the effects of mercury on the lungs are very limited. Alveolar type II epithelial cell and elastin damage were evident in the lung tissues of rats exposed to mercury via a similar pathway of oxidative stress and stimulation of the immune system (Koopsamy Naidoo et al. 2019) ^[64]^. The risk for mercury vapor was reported among mining workers (Eisler 2003). Acute respiratory distress, pneumothorax, and acute chemical pneumonitis were found after acute mercury exposure in a 3-month-old infant (Gao et al. 2017). However, there is no definite association between the impairment of lung function or prevalence of COPD and exposure to mercury (Rahman et al. 2022a, b; Pan et al. 2020). Our study showed that serum mercury was strongly correlated with a decline in FVC % predicted in all participants and current smokers, but the correlation with the prevalence of PRISm was not significant.

There are several limitations. First, in this cross-sectional study, we are unable to establish a temporal or causal link between the heavy metals and PRISm or lung function, including PRISm and COPD morbidity, COPD mortality, and lung function trajectory. Second, at the baseline test, PRISm was not detected using post-bronchodilator spirometry, which may lead to an overestimation of the prevalence of PRISm and COPD. However, the prevalence of PRISm in this study using prebronchodilator spirometry was similar to the previous cross-sectional prevalence, ranging from 4%-22.3% (Wan et al. 2018, 2022; Wijnant et al. 2020; Higbee et al. 2022; Xiao et al. 2021; Zhao et al. 2022). Third, we did not collect data related to different subgroups within PRISm, which subjects are clinically and genetically heterogeneous (Wan et al. 2018). Fourth, we did not collect urine data. Resents evidence that concentrations of heavy metals in urine, a perceived indicator of the total burden of heavy metals than heavy metals in blood, which are better reflect the level of long-term exposure (Yang et al. 2019). However, heavy metals in blood have been shown to have overlap and significant correlation with heavy metals in urine, especially exposure levels are relatively high (Adams and Newcomb 2014; Birgisdottir et al. 2013; Higashikawa et al. 2000). We also lacked information on factors that might complicate or modify the relationship between exposure to heavy metals and the outcomes of interest, such as dietary intake and other air pollutants.

## Conclusion

In summary, our study suggests that serum cadmium is associated with a higher risk of PRISm and lower lung function, with the most significant effect on FEV_1_/FVC in particular; lead and mercury exposure harms lung function in never/former smokers and current smokers, respectively. Furthermore, the term "PRISm" was first introduced in 2023 Global Initiative for Chronic Obstructive Lung Disease (GOLD) Report, which claims that individuals with PRISm should be considered as "patients" and receive care and treatment because they are symptomatic and/or have functional and/or structural abnormalities (Global Initiative for Chronic Obstructive Lung Disease 2023). Given that COPD is a major global public health concern, there is a strong demand for the discovery of modifiable risk factors for COPD prevention from the perspective of population medicine. Importantly, our study results further emphasize the significance of non-tobacco risk factors and primary prevention.

### Supplementary Information

Below is the link to the electronic supplementary material.

Supplementary file1 (DOCX 2935 KB)

Supplementary file2 (DOCX 29 KB)

## Data Availability

We examined publicly available datasets, which can be found here: https://wwwn.cdc.gov/nchs/nhanes/Default.aspx, accessed on 9 June 2022.
