# Supplementary material for: Associations between environmental heavy metals exposure and preserved ratio impaired spirometry in the U.S. adults
Source: Environ Sci Pollut Res Int. 2023 Sep 25;30(49):108274–87. doi: 10.1007/s11356-023-29688-y (PMC10611825; doi:10.1007/s11356-023-29688-y)
Supplement: Supplementary file 1 — Supplementary file1 (DOCX 2935 KB) [file 11356_2023_29688_MOESM1_ESM.docx]

**Supplementary Information**

**Figure**


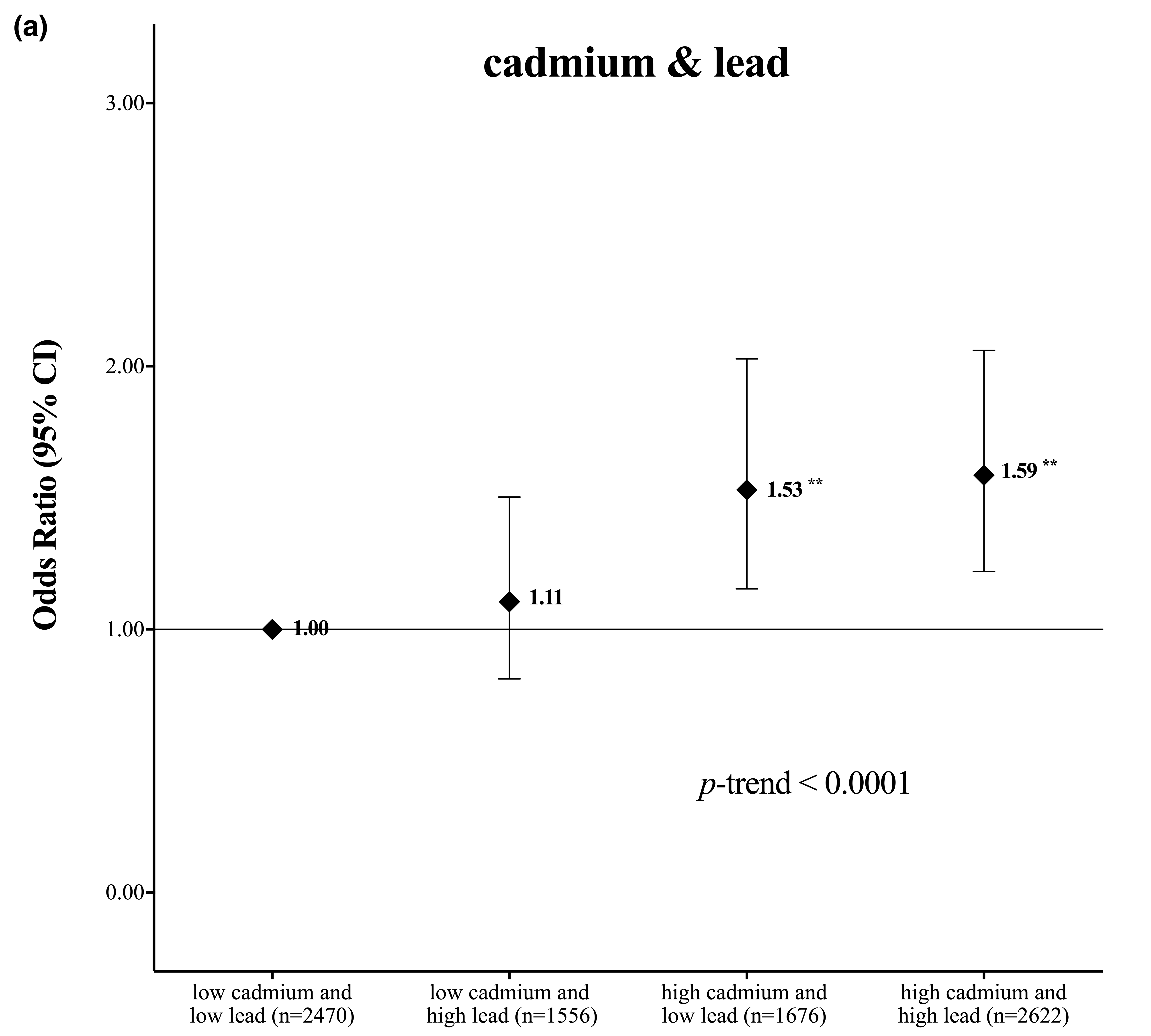


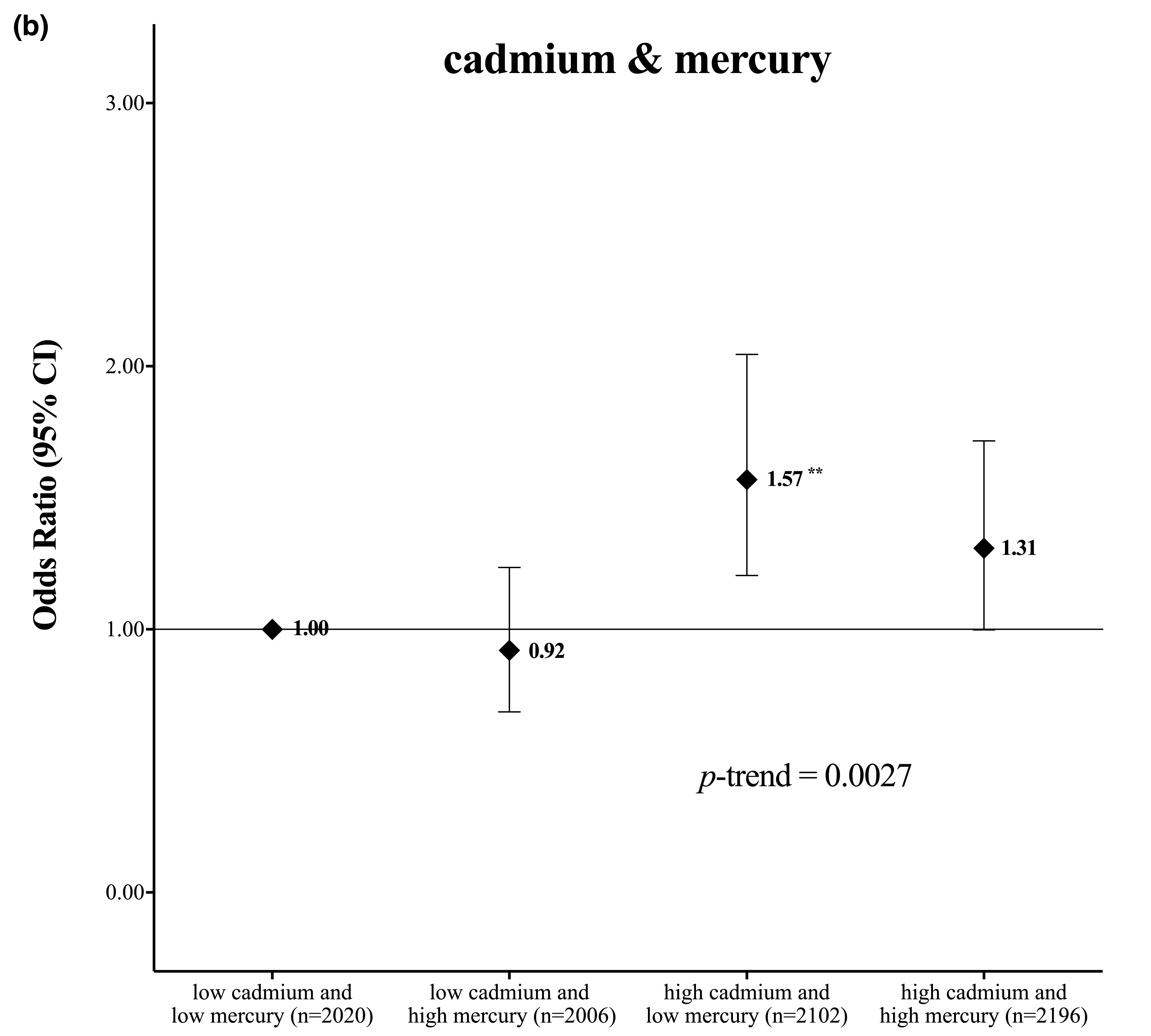


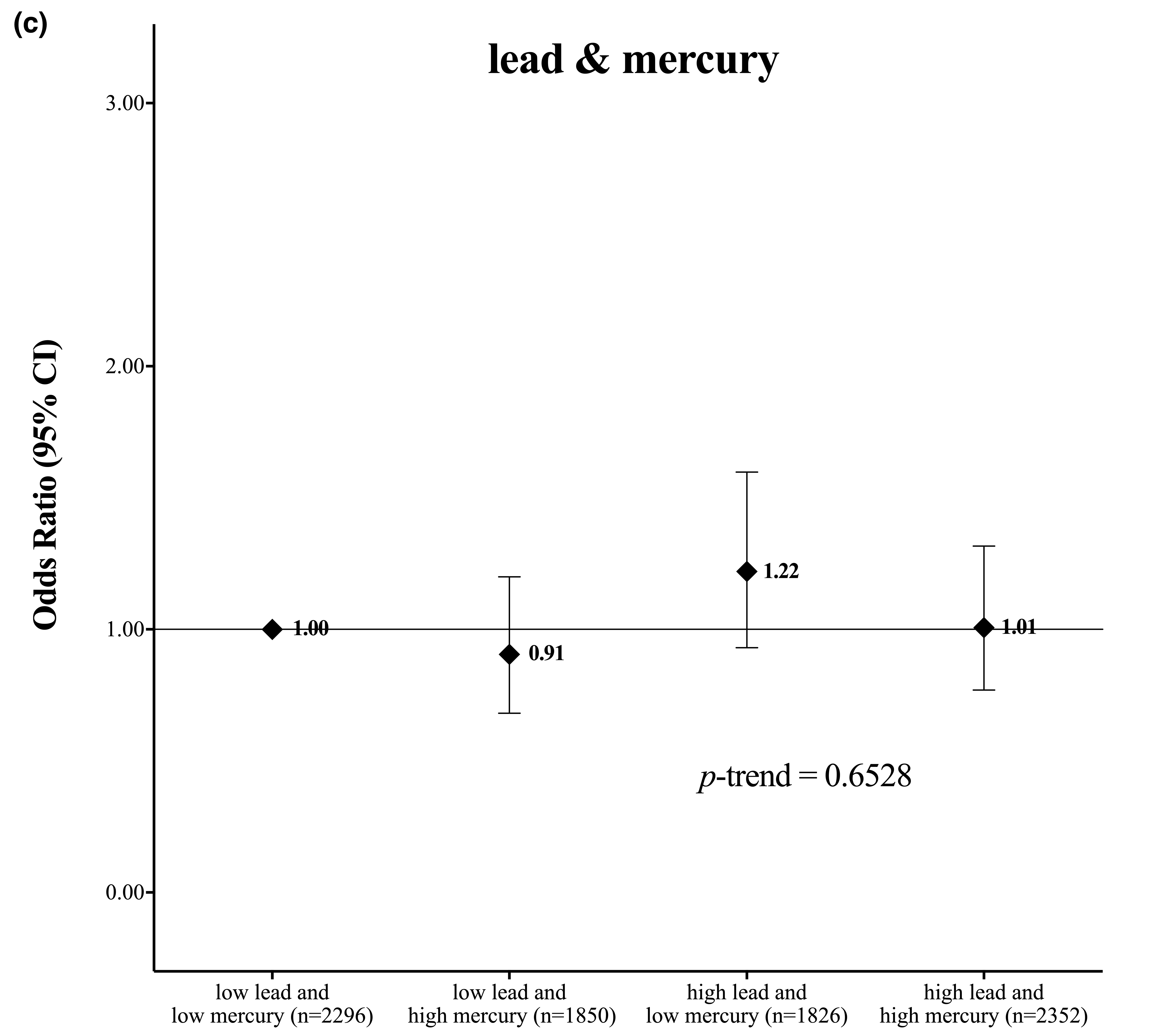


**Figure S1. Multivariate analyses of potential combined effects of cadmium, lead, and mercury levels on PRISm. (a)**, Combined effects of cadmium and lead. (**b)**, Combined effects of cadmium and mercury. (**c)**, Combined effects of lead and mercury. All models were adjusted: age, gender, race/ethnicity, BMI, PIR, health insurance, sedentary activity, history of childhood diseases (emphysema, bronchitis, or asthma), diabetes, and occupational exposure to mineral dusts, organic dusts or exhaust fumes. Serum concentrations of cadmium, lead, and mercury in the highest quartile (fourth) were regarded as high, whereas concentrations in the first to third quartiles were regarded as low. *p*-Value: ^**^ *p* < 0.01.
