# Supplementary material for: Associations between environmental heavy metals exposure and preserved ratio impaired spirometry in the U.S. adults
Source: Environ Sci Pollut Res Int. 2023 Sep 25;30(49):108274–87. doi: 10.1007/s11356-023-29688-y (PMC10611825; doi:10.1007/s11356-023-29688-y)
Supplement: Supplementary file 2 — Supplementary file2 (DOCX 29 KB) [file 11356_2023_29688_MOESM2_ESM.docx]

**Supplementary Information**

**Table**

**Table S1. Subgroup analysis of serum lead with PRISm^1^**

| **Lead** | **Sample Size** | **PRISm** | ***p*-Value** |
| --- | --- | --- | --- |
| Age group |  |  |  |
| <30 y | 2169 | 1.129 (1.033, 1.234) | 0.0072 |
| 30-39 y | 1631 | 1.019 (0.945, 1.098) | 0.6274 |
| 40-49 y | 1580 | 0.873 (0.698, 1.092) | 0.2353 |
| 50-59 y | 1291 | 0.941 (0.844, 1.050) | 0.2781 |
| 60-69 y | 1108 | 1.045 (0.935, 1.167) | 0.4370 |
| 70-79 y | 545 | 1.070 (0.847, 1.352) | 0.5684 |
| Sex |  |  |  |
| Female | 4200 | 0.929 (0.823, 1.048) | 0.2300 |
| Male | 4124 | 1.021 (0.976, 1.068) | 0.3639 |
| Race/ethnicity |  |  |  |
| Non-Hispanic White | 1884 | 0.928 (0.802, 1.073) | 0.3123 |
| Non-Hispanic Black | 4114 | 1.087 (1.010, 1.169) | 0.0260 |
| Mexican American | 2326 | 1.014 (0.945, 1.089) | 0.6924 |
| BMI (kg/m²) |  |  |  |
| < 18.5 (underweight) | 116 | 1.873 (0.954, 3.678) | 0.0684 |
| 18.5-24.9 (normal weight) | 2199 | 1.096 (1.010, 1.189) | 0.0287 |
| 25-29.9 (overweight) | 2714 | 0.984 (0.883, 1.096) | 0.7635 |
| ≥ 30 (obese) | 3288 | 0.982 (0.916, 1.054) | 0.6199 |
| Educational level |  |  |  |
| Primary school and less | 702 | 1.048 (0.966, 1.138) | 0.2609 |
| Middle and high school | 3011 | 0.973 (0.908, 1.043) | 0.4433 |
| College and higher | 4060 | 1.055 (0.924, 1.205) | 0.4285 |
| PIR |  |  |  |
| <1.85 | 3484 | 0.996 (0.941, 1.055) | 0.8952 |
| ≥1.85 | 4207 | 1.067 (0.977, 1.165) | 0.1481 |
| Health insurance coverage |  |  |  |
| No | 2349 | 0.984 (0.889, 1.088) | 0.7465 |
| Yes | 5964 | 1.016 (0.968, 1.066) | 0.5176 |
| Sedentary activity |  |  |  |
| <3h | 1704 | 0.973 (0.887, 1.068) | 0.5686 |
| 3-6h | 3092 | 1.033 (0.961, 1.111) | 0.3780 |
| ≥6h | 3517 | 1.038 (0.951, 1.133) | 0.4074 |
| Mineral dusts |  |  |  |
| No | 5410 | 0.975 (0.905, 1.050) | 0.5067 |
| Yes | 2540 | 1.059 (0.994, 1.128) | 0.0745 |
| Organic dusts |  |  |  |
| No | 6140 | 0.979 (0.913, 1.051) | 0.5592 |
| Yes | 1818 | 1.060 (0.997, 1.126) | 0.0631 |
| Fumes from machinery or engines |  |  |  |
| No | 5979 | 0.975 (0.912, 1.043) | 0.4583 |
| Yes | 1983 | 1.081 (1.009, 1.158) | 0.0272 |
| Any other gases, vapors or fumes |  |  |  |
| No | 5433 | 0.976 (0.908, 1.048) | 0.5031 |
| Yes | 2529 | 1.057 (0.990, 1.129) | 0.0977 |
| Emphysema, bronchitis or asthma during childhood |  |  |  |
| No | 6317 | 1.010 (0.962, 1.060) | 0.6862 |
| Yes | 2007 | 1.014 (0.909, 1.131) | 0.8008 |
| Emphysema |  |  |  |
| No | 7725 | 1.013 (0.970, 1.057) | 0.5657 |
| Yes | 50 | 0.133 (0.016, 1.103) | 0.0616 |
| Chronic bronchitis |  |  |  |
| No | 7450 | 1.007 (0.963, 1.054) | 0.7474 |
| Yes | 320 | 1.313 (0.928, 1.856) | 0.1239 |
| Asthma |  |  |  |
| No | 7283 | 1.012 (0.966, 1.060) | 0.6134 |
| Yes | 1035 | 1.005 (0.883, 1.144) | 0.9389 |
| Hypertension |  |  |  |
| No | 6032 | 1.045 (1.000, 1.091) | 0.0478 |
| Yes | 2283 | 0.884 (0.780, 1.002) | 0.0535 |
| Coronary heart disease |  |  |  |
| No | 7610 | 1.008 (0.964, 1.055) | 0.7188 |
| Yes | 155 | 0.995 (0.747, 1.325) | 0.9715 |
| Heart failure |  |  |  |
| No | 7660 | 1.011 (0.967, 1.057) | 0.6350 |
| Yes | 105 | 1.053 (0.816, 1.358) | 0.6931 |
| Stroke |  |  |  |
| No | 7628 | 1.013 (0.969, 1.058) | 0.5814 |
| Yes | 140 | 1.001 (0.757, 1.325) | 0.9922 |
| Diabetes |  |  |  |
| No | 7432 | 1.021 (0.978, 1.065) | 0.3519 |
| Yes | 748 | 0.926 (0.784, 1.093) | 0.3631 |
| Chronic cough |  |  |  |
| No | 4147 | 0.969 (0.904, 1.038) | 0.3690 |
| Yes | 372 | 1.025 (0.845, 1.243) | 0.8042 |
| Coughing phlegm |  |  |  |
| No | 4231 | 0.970 (0.906, 1.039) | 0.3887 |
| Yes | 291 | 1.032 (0.850, 1.254) | 0.7475 |
| Wheezing |  |  |  |
| No | 7386 | 1.022 (0.978, 1.068) | 0.3375 |
| Yes | 930 | 0.932 (0.791, 1.098) | 0.3984 |
| Shortness of breath |  |  |  |
| No | 3240 | 0.978 (0.890, 1.074) | 0.6386 |
| Yes | 1280 | 0.963 (0.878, 1.055) | 0.4156 |

Notes: BMI, body mass index; PIR: poverty income ratio; PRISm, preserved ratio impaired spirometry. ^1^ All models were adjusted: age, gender, race/ethnicity, BMI, PIR, health insurance, sedentary activity, history of childhood diseases (emphysema, bronchitis, or asthma), diabetes, and occupational exposure to mineral dusts, organic dusts or exhaust fumes. Subgroup analysis according to smoking status.

**Table S2. Subgroup analysis of serum mercury with PRISm**

| **Mercury** | **Sample Size** | **PRISm** | ***p*-Value** |
| --- | --- | --- | --- |
| Age group |  |  |  |
| <30 y | 2169 | 0.979 (0.807, 1.188) | 0.8316 |
| 30-39 y | 1631 | 0.999 (0.904, 1.104) | 0.9807 |
| 40-49 y | 1580 | 1.049 (0.984, 1.118) | 0.1410 |
| 50-59 y | 1291 | 1.060 (0.967, 1.162) | 0.2115 |
| 60-69 y | 1108 | 0.991 (0.898, 1.094) | 0.8623 |
| 70-79 y | 545 | 0.984 (0.831, 1.164) | 0.8466 |
| Sex |  |  |  |
| Female | 4200 | 1.015 (0.946, 1.089) | 0.6853 |
| Male | 4124 | 1.021 (0.971, 1.073) | 0.4134 |
| Race/ethnicity |  |  |  |
| Non-Hispanic White | 1884 | 0.981 (0.858, 1.122) | 0.7835 |
| Non-Hispanic Black | 4114 | 0.963 (0.890, 1.043) | 0.3543 |
| Mexican American | 2326 | 1.104 (1.037, 1.176) | 0.0021 |
| BMI (kg/m²) |  |  |  |
| < 18.5 (underweight) | 116 | 0.931 (0.540, 1.607) | 0.7984 |
| 18.5-24.9 (normal weight) | 2199 | 1.000 (0.900, 1.111) | 0.9949 |
| 25-29.9 (overweight) | 2714 | 1.021 (0.963, 1.084) | 0.4853 |
| ≥ 30 (obese) | 3288 | 1.036 (0.969, 1.109) | 0.3016 |
| Educational level |  |  |  |
| Primary school and less | 702 | 0.976 (0.827, 1.153) | 0.7784 |
| Middle and high school | 3011 | 1.046 (0.985, 1.111) | 0.1416 |
| College and higher | 4060 | 1.025 (0.968, 1.084) | 0.4020 |
| PIR |  |  |  |
| <1.85 | 3484 | 0.990 (0.909, 1.077) | 0.8105 |
| ≥1.85 | 4207 | 1.036 (0.991, 1.084) | 0.1202 |
| Health insurance coverage |  |  |  |
| No | 2349 | 0.989 (0.901, 1.086) | 0.8170 |
| Yes | 5964 | 1.036 (0.991, 1.084) | 0.1219 |
| Sedentary activity |  |  |  |
| <3h | 1704 | 0.986 (0.870, 1.118) | 0.8258 |
| 3-6h | 3092 | 1.045 (0.989, 1.105) | 0.1176 |
| ≥6h | 3517 | 1.002 (0.940, 1.067) | 0.9603 |
| Mineral dusts |  |  |  |
| No | 5410 | 1.027 (0.976, 1.080) | 0.3038 |
| Yes | 2540 | 1.005 (0.933, 1.083) | 0.8960 |
| Organic dusts |  |  |  |
| No | 6140 | 1.026 (0.982, 1.072) | 0.2516 |
| Yes | 1818 | 0.983 (0.865, 1.117) | 0.7957 |
| Fumes from machinery or engines |  |  |  |
| No | 5979 | 1.002 (0.950, 1.057) | 0.9400 |
| Yes | 1983 | 1.053 (0.989, 1.122) | 0.1086 |
| Any other gases, vapors or fumes |  |  |  |
| No | 5433 | 1.031 (0.980, 1.084) | 0.2426 |
| Yes | 2529 | 1.005 (0.933, 1.082) | 0.8980 |
| Emphysema, bronchitis or asthma during childhood |  |  |  |
| No | 6317 | 1.033 (0.990, 1.078) | 0.1370 |
| Yes | 2007 | 0.969 (0.858, 1.094) | 0.6069 |
| Emphysema |  |  |  |
| No | 7725 | 1.021 (0.981, 1.062) | 0.3107 |
| Yes | 50 | 0.375 (0.008, 16.938) | 0.6141 |
| Chronic bronchitis |  |  |  |
| No | 7450 | 1.020 (0.978, 1.063) | 0.3545 |
| Yes | 320 | 1.055 (0.880, 1.264) | 0.5635 |
| Asthma |  |  |  |
| No | 7283 | 1.026 (0.984, 1.070) | 0.2260 |
| Yes | 1035 | 0.969 (0.830, 1.131) | 0.6879 |
| Hypertension |  |  |  |
| No | 6032 | 0.972 (0.910, 1.037) | 0.3895 |
| Yes | 2283 | 1.112 (1.039, 1.191) | 0.0021 |
| Coronary heart disease |  |  |  |
| No | 7610 | 1.021 (0.981, 1.064) | 0.3027 |
| Yes | 155 | 0.962 (0.725, 1.276) | 0.7884 |
| Heart failure |  |  |  |
| No | 7660 | 1.025 (0.985, 1.066) | 0.2273 |
| Yes | 105 | 0.820 (0.565, 1.189) | 0.2949 |
| Stroke |  |  |  |
| No | 7628 | 1.022 (0.982, 1.064) | 0.2791 |
| Yes | 140 | 0.894 (0.450, 1.775) | 0.7483 |
| Diabetes |  |  |  |
| No | 7432 | 1.024 (0.983, 1.066) | 0.2612 |
| Yes | 748 | 0.985 (0.848, 1.145) | 0.8440 |
| Chronic cough |  |  |  |
| No | 4147 | 1.027 (0.980, 1.077) | 0.2590 |
| Yes | 372 | 1.080 (0.841, 1.386) | 0.5477 |
| Coughing phlegm |  |  |  |
| No | 4231 | 1.030 (0.983, 1.079) | 0.2113 |
| Yes | 291 | 1.032 (0.776, 1.372) | 0.8288 |
| Wheezing |  |  |  |
| No | 7386 | 1.033 (0.992, 1.076) | 0.1176 |
| Yes | 930 | 0.913 (0.764, 1.090) | 0.3125 |
| Shortness of breath |  |  |  |
| No | 3240 | 1.029 (0.975, 1.085) | 0.2953 |
| Yes | 1280 | 1.082 (0.980, 1.195) | 0.1201 |

Notes: BMI, body mass index; PIR: poverty income ratio; PRISm, preserved ratio impaired spirometry. ^1^ All models were adjusted: age, gender, race/ethnicity, BMI, PIR, health insurance, sedentary activity, history of childhood diseases (emphysema, bronchitis, or asthma), diabetes, and occupational exposure to mineral dusts, organic dusts or exhaust fumes. Subgroup analysis according to smoking status.
